# Draft Genome Sequences for the *Frankia* sp. strains CgS1, CcI156 and CgMI4, Nitrogen-Fixing Bacteria Isolated from *Casuarina* sp. in Egypt

**DOI:** 10.7150/jgen.51181

**Published:** 2020-09-23

**Authors:** Samira Mansour, Erik Swanson, Céline Pesce, Stephen Simpson, Krystalynne Morris, W. Kelley Thomas, Louis S. Tisa

**Affiliations:** 1 Faculty of Science, Suez Canal University, Ismailia, Egypt; 2 University of New Hampshire, Durham, New Hampshire, USA; 3 Present address: HM Clause, Davis, California, USA

**Keywords:** actinobacteria, actinorhizal symbiosis, genomes, host microbe interactions, nitrogen fixation, natural products, salt tolerance

## Abstract

*Frankia* sp. strains CgS1, CcI156 and CgMI4 were isolated from *Casuarina glauca* and C. *cunninghamiana* nodules. Here, we report the 5.26-, 5.33- and 5.20-Mbp draft genome sequences of *Frankia* sp. strains CgS1, CcI156 and CgMI4, respectively. Analysis of the genome revealed the presence of high numbers of secondary metabolic biosynthetic gene clusters.

## Introduction

Members of the genus *Frankia* are soil-dwelling Actinobacteria that form a symbiotic association with Angiosperms, representing eight different plant families 1-3. This plant-microbe association, termed actinorhizal, aid these pioneer plants in colonizing harsh environments and disrupted lands 2, 4, 5. Actinorhizal plants play a role economically in agroforestry, land reclamation, crop protection, and soil stabilization projects 4-7. Based on molecular phylogeny, *Frankia* strains group into four major clusters that also follow plant host range specificity 8-11. Cluster 1 consists of *Frankia* strains that associate with host plants in the Casuarinaceae, Betulaceae and Myricaceae families, while members of cluster 2 are infective on Rosaceae, Coriariaceae, Datiscaceae, and the genus *Ceanothus* (Rhamnaceae). Cluster 3 are the most promiscuous and are infective on Elaeagnaceae, Rhamnaceae, Myricaceae, *Gynmnostoma*, and occasionally the genus *Alnus.* Cluster 4 consists of “atypical” *Frankia* strains that are unable to re-infect actinorhizal host plants or form ineffective nonnitrogen-fixing root nodule structures.

Representative genomes from each cluster have been sequenced 12. These *Frankia* genome databases has enabled the use of “omics” approaches 13-15 and allowed speciation of the genus 16.

Cluster 1 is further divided into subclades with subclade Ic containing strains limited to *Casuarina* and *Allocasuarina* and Myricaceae host plants. Actinorhizal plants belonging to *Casuarinaceae* family such as *Casuarina glauca* and *C. equisetifolia* will grow under saline environments 4, 5, 13. These fast-growing trees originated from Australia and Pacific islands, and are widely used in agroforestry systems for several purposes 17. Large-scale planting of casuarinas has proven to have a strong impact especially in China, Senegal, Egypt and Tunisia 18. In arid and semi-arid areas, salinization of soils and groundwater is a serious problem causing a drastic reduction in agricultural production (19, 20; http://www.fao.org/nr/land/degradation/en/). Over 800 million hectares of land throughout the world are salt-affected 20. An effective method for the reclamation of salt-affected soils involves initiating plant succession using fast growing, nitrogen fixing actinorhizal trees such as the Casuarina. The salt tolerance of *Casuarina* is enhanced by the nitrogen-fixing symbiosis that they form with *Frankia*.^7^. A broad spectrum “omics” approach on *Frankia* strains from *Casuarina* plants has allowed us to define several genes involved salt tolerance 21 including one gene that was cloned into a salt-sensitive strain 22. The expression of the cloned genes provided increased tolerance to salt in the receipt strain. Here we have isolated three *Frankia* strains from *Casuarina* species that were found under salt stress conditions. These strains were sequenced to provide more information about salt tolerance genes and a greater understanding the metabolic potential of these actinobacteria.

## Isolation of Frankia strains

**Figure 1:**
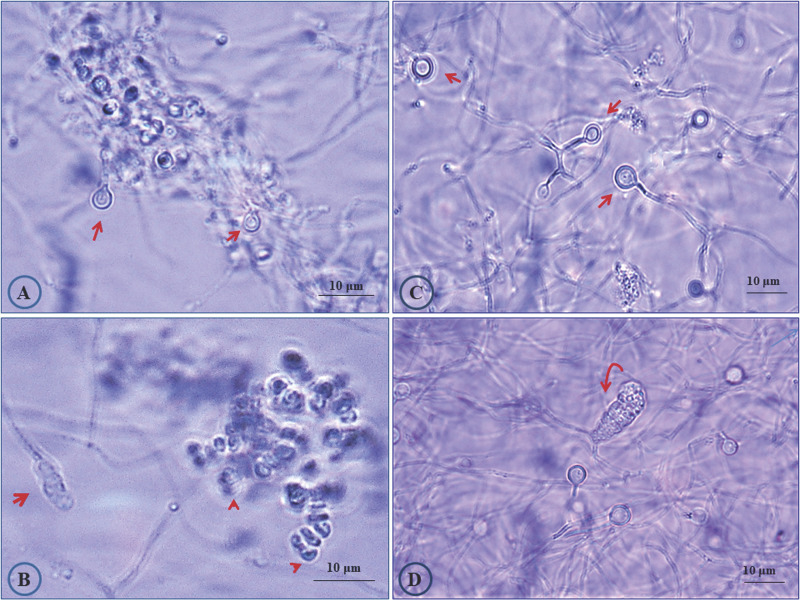
Photomicrograph showing morphological features of *Frankia* sp. strains CgIM4 and CcI156. Cultures were grown in liquid B medium 23 for 45 days. (A) *Frankia* sp. strain CgIM4 showing typical active vesicles with double wall (arrow); (B) sporulation of *Frankia* sp. strain CgIM4 forming typical multilocular sporangium (arrow) and the release of mature spores (head arrows); (C) healthy vesicles (arrows) for *Frankia* sp. strain CcI156; (D) *Frankia* sp. strain CcI156 intercalary multilocular sporangium with acropetal spore maturation (arrow).

Three different *Casuarina* plantation sites in Egypt were chosen for sampling and root nodules were collected and transferred to the laboratory in an icebox. From site one, the nodule sample was collected from a large *Casuarina glauca*. tree growing in sandy loamy soil around a Mango Garden, as “green-barrier fence, in Abuo-Shata, Ismailia, Egypt. For the second site, nodules were collected from *Casuarina cunninghamiana* tree growing inside a nearby Mango garden in Abuo-Shata, Ismailia, Egypt (Latitude and longitude coordinates: 30°35′47.37″N, 32°16′17.25″E). The third nodule sample was collected from *C. glauca* growing in sandy soil along Ismailia-Cairo highway (30.0444° N, 31.2357° E). After the nodules were cleaned with tap water and surface sterilized, *Frankia* strains were isolated as previously described 23. *Frankia* isolates obtained from site 1, 2 and 3 were designated CgIM4, CcI156 and CgIS1, respectively, and propagated in B medium 23. The *Frankia* isolates displayed the typical morphological traits of the genus *Frankia* (Fig. 1). These developmentally complex bacteria form three cell types: vegetative hyphae, spores located in sporangia and the unique lipid-enveloped cellular structures, termed vesicles 1, 3. Vesicles act as specialized structures for the nitrogen fixation process 1, 3.

**Figure 2:**
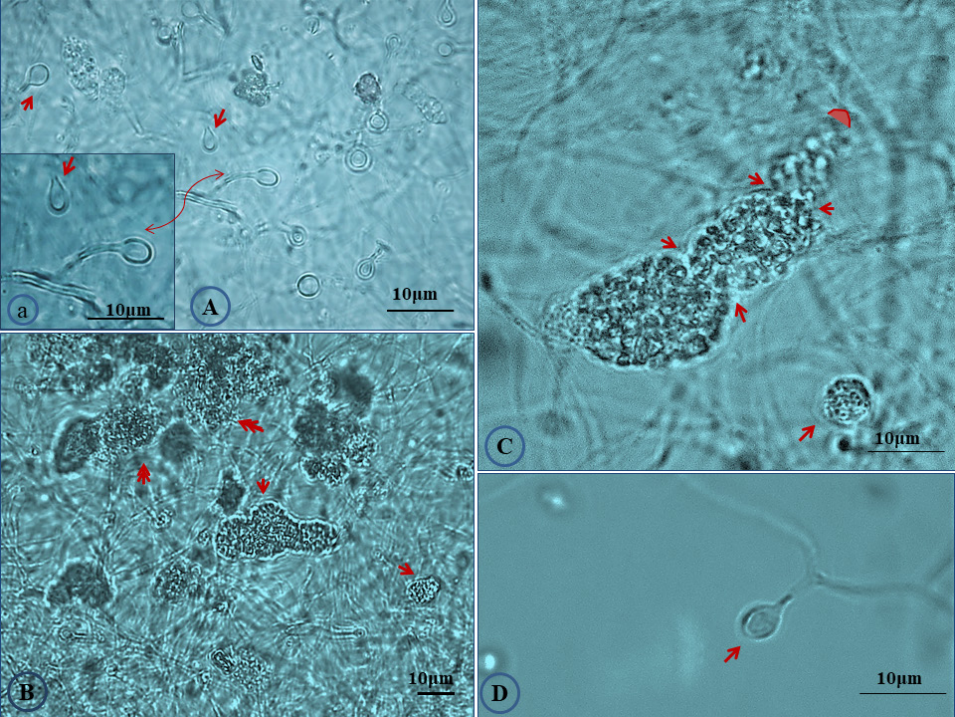
Effect of salt stress on the morphological characters of *Frankia* sp. strain CgIM4. Cultures were grown in B medium 23 under 300 mM NaCl stress for 45 days. (A) Vesicles showed slight deformation in which the elliptical shape with thinner wall was observed (arrow) compared to thick circular shape in normal condition; (a) magnified section of panel A showing deformed vesicles; (B) abundance of mature sporangia (arrows) were detected at a high frequency; (C) twisted mature sporangia (arrows) with constriction which showed mature spores filling the whole sporangium, note the absence of acropetal spore maturation as under non-stressed conditions (Fig. 1); (D) deformed vesicle.

The salt tolerance levels of *Frankia* sp. strains CcI156 and CgMI4 were determined by measuring the total cellular protein content after growth under salt stress, as described previously 21. *Frankia* sp. strain CcI6, a salt-tolerant strain, and *Frankia casuarinae* strain CcI3, a salt-sensitive strain, were included as controls (Fig. S1). *Frankia* sp. strains CcI156 and CgMI4 have MIC values of 550 and 700 mM NaCl, respectively. *Frankia* sp. strain CcI6 and *F. casuarinae* strain CcI3 have MIC values of 1,000 and 475 mM NaCl, respectively, similar to previous results 21. The MIC value for *Frankia* sp. strains CcI156 was similar to the values obtained for other *Frankia* lineage Ic strain (*Frankia* sp. strains BR, BMG5.23, CeD and Thr), while *Frankia* sp. strain CgMI4 was greater than those strains but not to the level of *Frankia* sp. strains CcI6 and Allo2 21. *Frankia* sp. strains CgMI4 and CcI156 exhibited several morphological changes when grown under salt-stress conditions (Fig. 2; Fig S2). Both sporangium and vesicle development were altered.

## Sequencing of Frankia strains CgS1, CcI156 and CgMI4

Sequencing of the draft genomes of *Frankia* sp. strains CgS1, CcI156 and CgMI4 was performed at the Hubbard Center for Genome Studies (University of New Hampshire, Durham, NH) using Illumina technology techniques 24. For each genome, a standard Illumina shotgun library was constructed and sequenced using the Illumina HiSeq2500 platform, using a pair-end library with an average size of 600 bp obtaining 5,345,232, 11,011,806 and 11,746,204 reads of 250 bp in length for strains CgS1, CcI156 and CgMI4, respectively (Table 1). The Illumina sequence data were trimmed by Trimmonatic version 0.32 25, assembled using Spades version 3.5 25 and ALLPaths-LG version r52488 26. The metrics for final draft assemblies for *Frankia* sp. strains CgS1, CcI156 and CgMI4 are found in Table 1. The two 135 and 145 contigs ranged from 5.2 to 5.3 Mbp in size with 70% G + C content.

The assembled genomes were annotated *via* the NCBI Prokaryotic Genome Annotation Pipeline (PGAP) 27. The annotation features are also given in Table 1. The genome features of three *Frankia* sp. strains are similar to other cluster 1c genomes (Table 1) including *F. casuarinae* strain CcI3^T^ 28. Analysis of the three genomes for the number of genes associated with the Clusters of Orthologous Groups (COG) functional categories showed that the pattern of distribution was similar to *F. casuarinae* strain CcI3^T^ (Table S1). Phylogenetic analysis of the 16S rDNA shows that *Frankia* sp. strains CgS1, CcI156 and CgMI4 group with the cluster 1c strains (Figure S3) and further confirmed by dendrogram of the entire genomes (Figure S4). A whole genome-based taxonomic analysis via the Type (Strain) Genome Server (TYGS) platform 29 (https://tygs.dsmz.de) including digital DNA:DNA hybridization (dDDH) values 30 was performed to identify these strain compared to other *Frankia* genomes from Cluster 1c (Table S2). The type-based species clustering using a 70% dDDH radius around each of the type strains was used as previously 31, while subspecies clustering was done using a 79% dDDH threshold as previously introduced 32. These data indicate that *Frankia* sp. strains CgS1, CcI156 and CgMI4 are members of *Frankia casuarinae* species. Average nucleotide identity (ANI) analysis of these genomes (Table S3) confirmed that idea with an ANI threshold range of 95% for species delineation 33.

Bioinformatic analysis of this genomes by the use of the AntiSMASH program 34 revealed the presence of high numbers of secondary metabolic biosynthetic gene clusters (Table 2), which is consistent with previous results with other *Frankia* lineage 3 genomes including cluster 3 12, 35. Many of these potential natural products should be involved in the plant-microbe interactions and aiding in their plant-growth-promoting activities. Analysis of the *Frankia* sp. strain CgS1, CcI156 and CgMI4 genomes revealed similar salt-tolerance genes as previously identified including several hypothetical proteins responsive to salt stress detected by genomic, transcriptomic, and proteomic approaches 21.

In summary, the *Frankia* sp. strain CgS1, CcI156 and CgMI4 genome has revealed an interesting metabolic potential including secondary metabolites pathways and natural product profiles and serves as more representatives of *Frankia* cluster 1c.

### Nucleotide sequence accession numbers

These whole-genome shotgun sequences have been deposited at DDBJ/EMBL/GenBank under the accession numbers: MBLP00000000, MOMD00000000 and MAQZ00000000 for strains CgS1, CcI156 and CgMI4, respectively. The versions described in this paper are the first versions, MBLP 01000000, MOMD01000000, and MAQZ01000000 for strains CgS1, CcI156 and CgMI4, respectively. Both the assembly and raw reads are available at DDBJ/ENA/GenBank under BioProject number PRJNA299691, PRJNA299689, and PRJNA286879 for strains CgS1, CcI156 and CgMI4, respectively.

## Supplementary Material

Supplementary figures and tables.

## Figures and Tables

**Table 1:** Genome features of *Frankia* sp. strain CgIS1, CcI156 and CgMI4 isolated from *Casuarina* root nodules.

Strain	CgIS1	CcI156	CgMI4
Reads	5,345,232	11,011,806	11,746,204
N_50_ (kb)	99.0	80.6	90.5
Contigs	135	145	135
Genome size	5,257,145	5,330,592	5,199,090
Coverage	157.9X	427.0X	328.1X
G + C content (%)	70.03	69.90	69.97
CDS (coding)	4,224	4,304	4,165
rRNA	2	2	0
tRNA	45	45	46
GeneBank Accession number	MBLP00000000	MOMD00000000	MAQZ00000000
BioProject	PRJNA299691	PRJNA299689	PRJNA286879

**Table 2:** Biosynthetic gene clusters (BGS) for natural products found in the select *Frankia* genomes

Strain	CgS1	CcI156	CgMI4	CcI6	CcI3
Total BGC Clusters	30	35	33	33	29
NRPS^1^	6	9	7	8	3
PKS^2^	5	8	8	8	5
Terpene	4	4	4	4	4
Siderophore	1	1	1	1	1
Bacteriocin Lassopeptide	2	2	2	3	3
Lantipeptide	5	5	6	5	6
Reference	This study	This study	This study	36	37

Biosynthettic gene clusters were identified by the use of the AntiSMASH software 34. ^1^ NRPS: Nonribosomal peptide synthase. ^2^ PKS: polyketide synthase including Type I, II, III, Trans-AT and other types
