# Supplementary material for: Draft Genome Sequences for the Frankia sp. strains CgS1, CcI156 and CgMI4, Nitrogen-Fixing Bacteria Isolated from Casuarina sp. in Egypt
Source: J Genomics. 2020 Sep 23;8:84–8. doi: 10.7150/jgen.51181 (PMC7532629; doi:10.7150/jgen.51181)
Supplement: Supplementary file 1 — Supplementary figures and tables. [file jgenv08p0084s1.pdf]

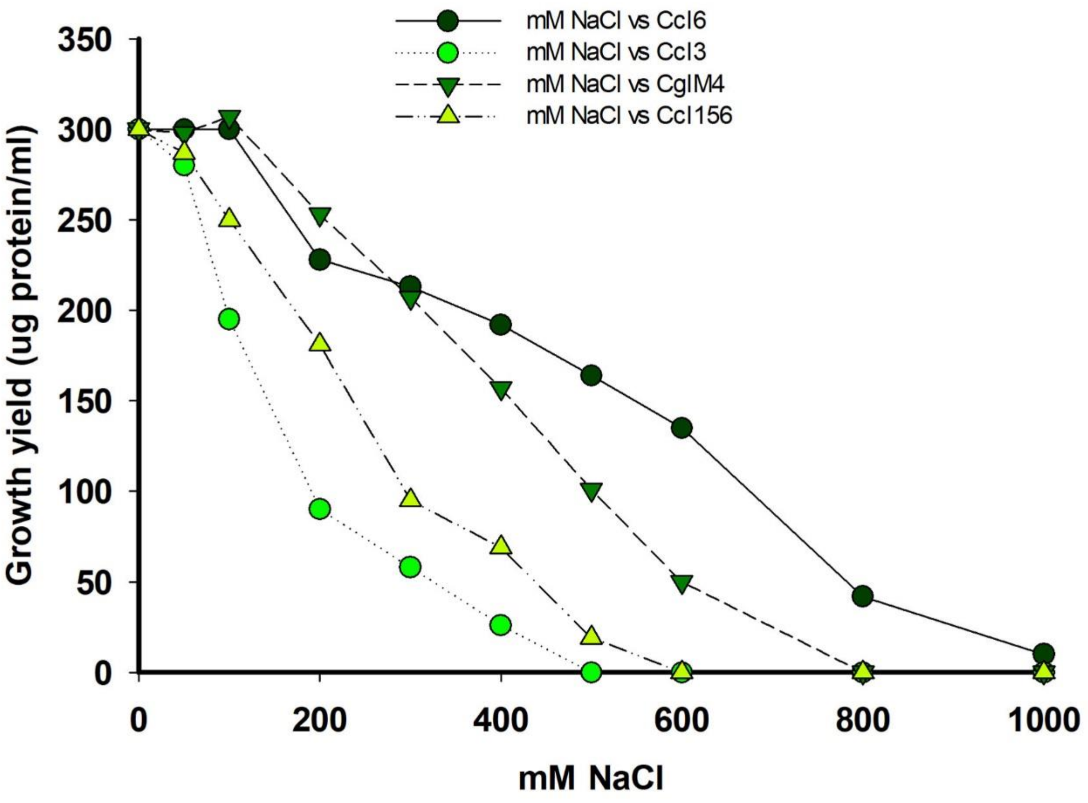

Figure S1. Salt sensitivity assay for *Frankia* strains. *Frankia* cultures were grown for 30 days in growth medium containing different NaCl concentrations. The growth yield was determined by measuring total protein content and corrected for inoculum content. Growth curves were standardized to 300 ug/ml for the control conditions (0 mM NaCl). Symbols represent *F. casuarinae* strain CcI3 (closed green circles), *Frankia* sp, strain CcI6 (closed black circles), Frankia sp. strain CgIM4 (inverted triangles), and *Frankia* sp. strain CcI156 (green triangles)

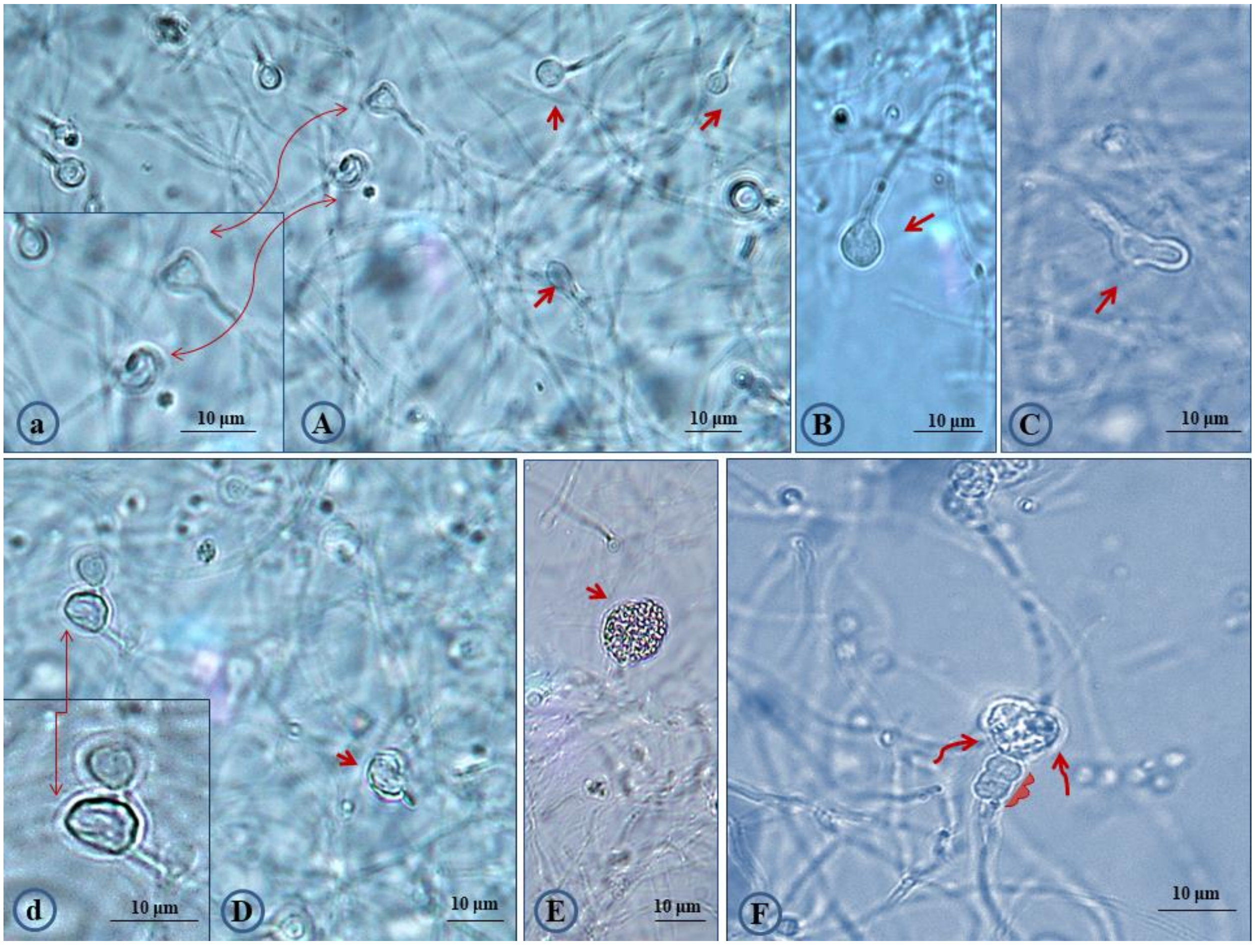

Figure S2. Effect of salt stress on the morphological characters of *Frankia* sp. strain CcI156. Cultures were grown in B medium [22] under 300 mM NaCl stress for 45 days. (A) vesicle deformation observed forming different shapes (arrows); (a) magnified section of panel A showing deformed vesicles showing cup-shape and inverted G-shape (long arrows); (B) thin-walled dumpy pear-shape vesicle; (C) peanut-shape deformed vesicle with double active wall; (D) an irregular and wrinkled thin-wall odd-shaped vesicle; (d), magnified section of panel D showing a deformed vesicle with a wrinkled wall (long arrow); (E) sub-spherical mature multilocular-enclosed sporangium that is full with mature spores (arrow); (F) an irregular-shaped sporangium with an oblong base that ends with a heart-shape top (arrows).

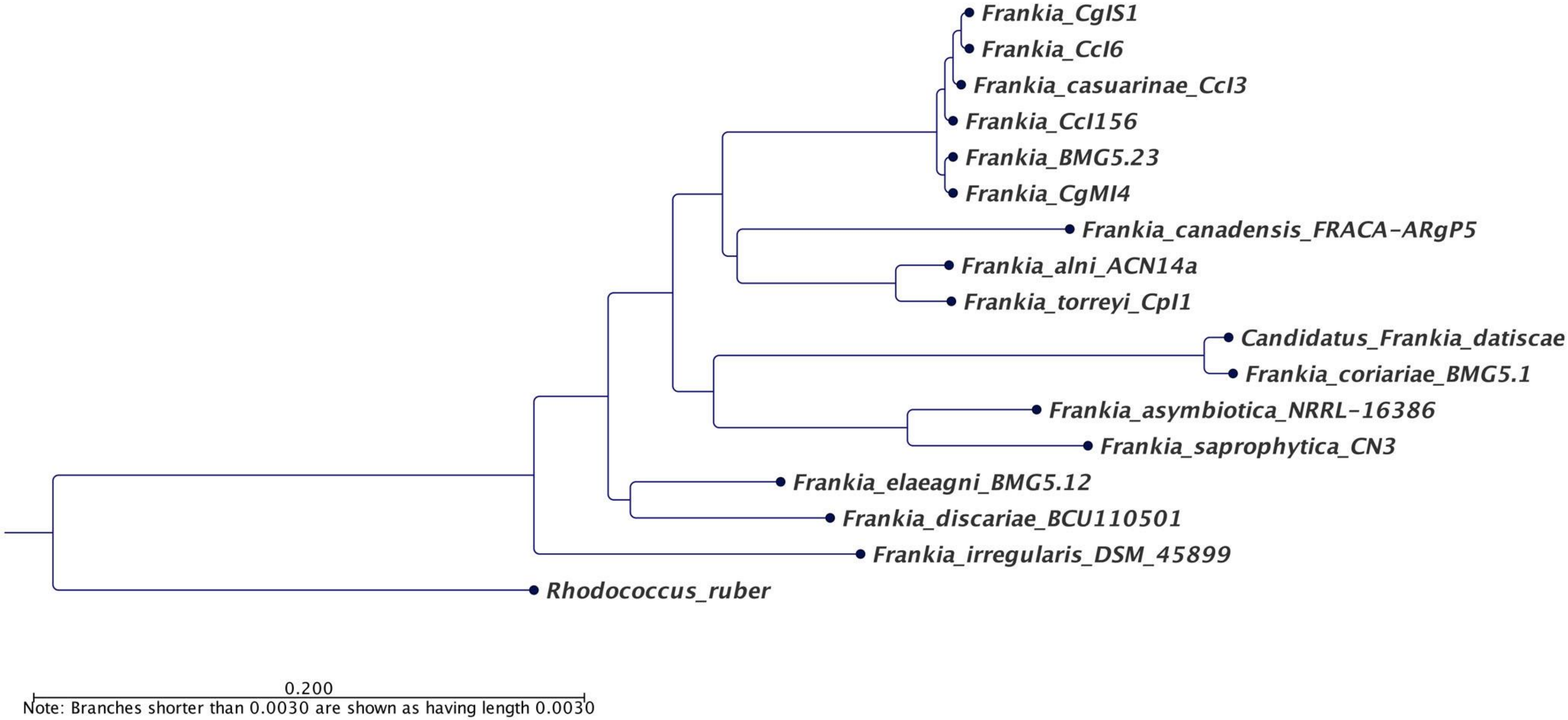

Figure S3. Neighbor-joining phylogenetic tree based on 16SrRNA sequences showing the position of *Frankia* sp. strains CgMI4, CgIS1 and CcI156 within the genus *Frankia* and an outgroup. The tree was rooted at the midpoint.

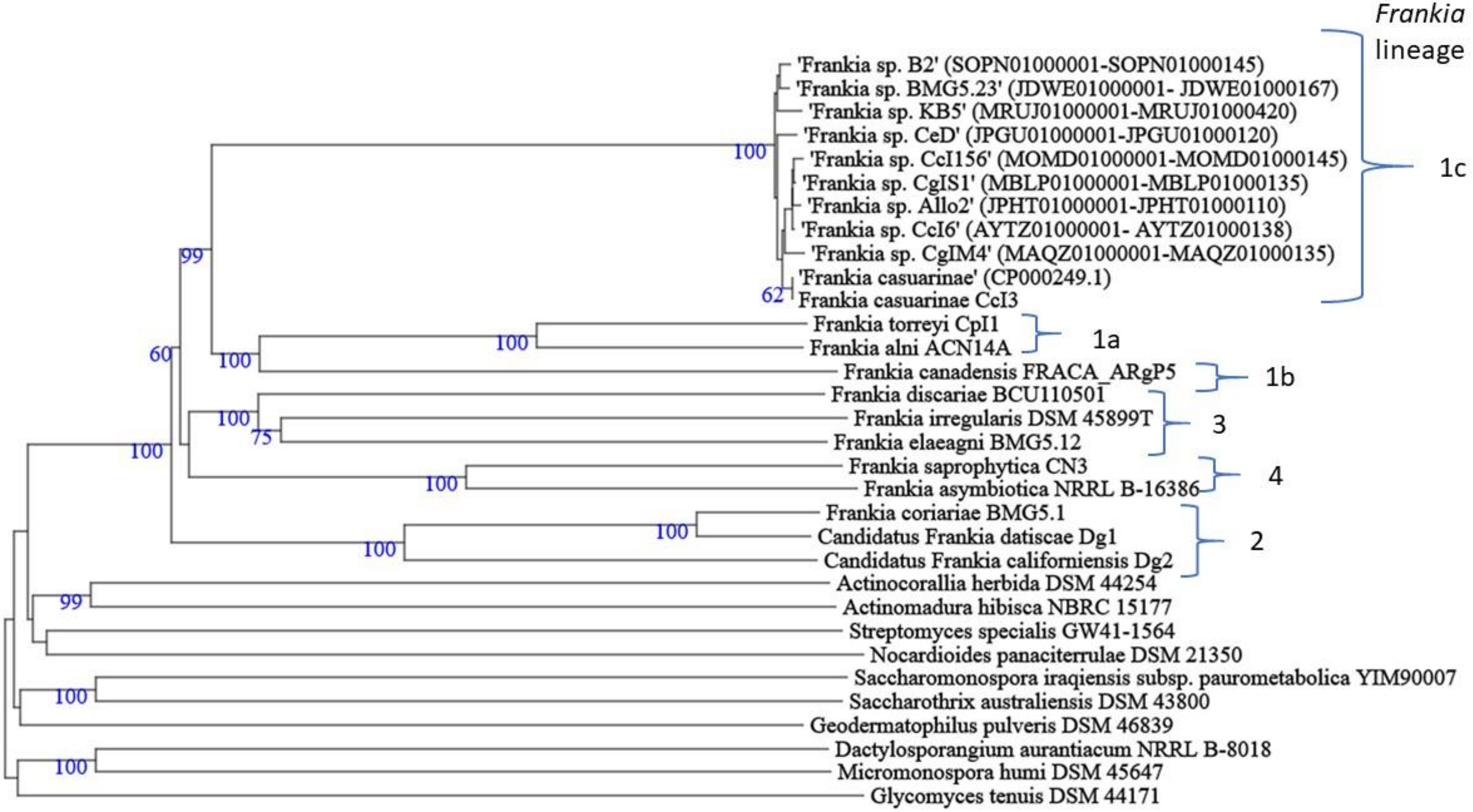

Figure S4. Tree inferred with FastME 2.1.6.1 from Genome BLAST Distance Phylogeny (GBDP) distances calculated from genome sequences. The branch lengths are scaled in terms of GBDP distance formula *d5*. The numbers above branches are GBDP pseudo-bootstrap support values > 60 % from 100 replications, with an average branch support of 59.0 %. The tree was rooted at the midpoint. *Frankia* lineages identified by brackets.

Table S1. Number of genes associated with the Clusters of Orthologous Groups (COG) functional categories

COG group	*Frankia* sp. CgIM4	*Frankia* sp. CgIS1	*Frankia* sp. CcI156	*Frankia casuarinae* CcI3
**Cellular processes**				
[D] Cell cycle control, cell division, chromosome partitioning	27 (1.03%)	29 (1.06%)	29 (1.05%)	29 (1.06%)
[M] Cell wall/membrane/envelope biogenesis	146 (5.56%)	156 (5.71%)	158 (5.73%)	156 (5.65%)
[N] Cell motility	16 (0.61 %)	16 (0.58%)	17 (0.62%)	16 (0.58%)
[O] Posttranslational modification, protein turnover, chaperones	130 (4.95%)	133 (4.87%)	134 (4.86%)	125 (4.58%)
[T] Signal transduction mechanisms	164 (5.86%)	155 (5.68%)	158 (5.73%)	158 (5.76%)
[U] Intracellular trafficking, secretion, and vesicular transport	21 (0.8%)	22 (0.81%)	22 (0.80%)	25 (0.91%)
[V] Defense mechanisms	70 (2.66%)	76 (2.78%)	76 (2.78%)	70 (2.55%)
[W] Extracellular structures	4 (0.15%)	4 (0.15%)	4 (0.15%))	7 (0.26%)
[Y] Nuclear structure	0	0	0	0
[Z] Cytoskeleton	0	0	0	0
**Information storage and processing**				
[A] RNA processing and modification	1 (0.04%)	1 (0.04%)	1 (0.04%)	1 (0.04%)
[B] Chromatin structure and dynamics	1 (0.04%)	1 (0.04%)	1 (0.04%)	1 (0.04%)
[L] Replication, recombination and repair	97 (3.69%)	108 (3.55%)	107 (3.88%)	111 (4.05%)
[K] Transcription	188 (6.77%)	206 (7.54%)	207 (7.51%)	192 (7.00%)
[J] Translation, ribosomal structure and biogenesis	178 (6.77%)	184 (6.74%)	184 (6.67%)	181 (6.60%)
[X] Mobilome: prophages, transposons	21 (0.08%)	39 (1.43%)	36 (1.31%)	73 (2.66%)
**Metabolism**				
[C] Energy production and conversion	185 (7.04%)	180 (6.59%)	184 (6.67%)	192 (7.00%)
[E] Amino acid transport and metabolism	205 (7.8%)	207 (7.58%)	211 (7.65%)	210 (7.66%)

[F] Nucleotide transport and metabolism	80 (3.04%)	87 (3.19%)	87 (3.15%)	90 (3.23%)
[G] Carbohydrate transport and metabolism	139 (5.29%)	141 (5.16%)	141 (5.11%)	139 (5.07%)
[H] Coenzyme transport and metabolism	187 (7.12%)	195 (7.14%)	195 (7.07%)	199 (7.26%)
[I] Lipid transport and metabolism	169 (6.43%)	164 (6.01%)	170 (6.16%)	167 (6.09%)
[P] Inorganic ion transport and metabolism	123 (4.68%)	126 (4.61%)	129 (4.68%)	129 (4.70%)
[Q] Secondary metabolites biosynthesis, transport and catabolism	113 (4.3%)	114 (4.17%)	117 (4.24%)	106 (3.87%)
**Poorly Characterized**				
[S] Function unknown	103 (3.92%)	103 (3.77%)	105 (3.81%)	97 (3.54%)
[R] General function prediction only	270 (10.27%)	284 (10.4%)	285 (10.33%)	269 (9.81%)
Not in COG	1851 (43.94%)	2360 (48.81%)	2408 (49.13%)	2183 (47.24%)

Table S2. Digital DNA:DNA hybridization (dDDH) values for *Frankia* isolates from Lineage 1c and selected other lineages.

**Genome 2**	**CgIM4**	**CgS1**	**CcI156**	**CcI3**	**CcI6**	**BMG5.23**	**B2**	**Allo2**	**CeD**	**KB5**
Frankia casuarinae CcI3	94.6	96.0	94.8	-	96.3	94.7	94.2	95.2	94.6	92.8
Frankia sp. CcI6	95.9	99.3	98.6	96.3	-	94.7	94.6	98.9	94.0	92.4
Frankia sp. BMGG5.23	93.6	94.4	93.3	94.7	94.7	-	96.4	93.7	94.4	94.3
Frankia sp. KB5	91.3	92.1	91.0	92.9	92.4	94.3	98.8	92.1	93.1	92.1
Frankia sp. B2	93.2	94.3	93.3	94.2	94.6	96.4	-	93.6	94.5	93.8
Frankia sp. Allo2	95.0	98.9	97.9	95.2	98.9	93.7	93.6	-	94.0	91.3
Frankia sp. CeD	93.2	93.8	92.7	94.6	94.0	94.4	94.5	94.0	-	93.1
Frankia sp. CcI156	97.7	98.6	-	94.8	98.6	93.3	93.3	97.9	92.7	91.0
Frankia sp. CgIS1	95.7	-	98.6	96.0	99.3	94.4	94.3	98.9	93.8	92.1
Frankia sp. CgIM4	-	95.7	94.7	94.6	95.9	93.6	93.2	95.0	93.2	91.3
Frankia alni ACN14a	25.0	25.3	25.1	25.4	25.2	25.2	25.2	25.0	25.1	25.1
Frankia coriariae BMG5.1	23.6	23.7	23.6	24.0	23.7	23.7	23.4	23.7	23.9	23.6
Frankia elaeangi BMG5.12	23.2	23.4	23.4	23.4	23.5	23.3	23.5	23.4	23.2	23.5
Frankia saprophytica CN3	22.6	22.8	22.7	23.0	22.9	23.0	23.0	22.7	22.8	22.6

dDDH values were determined using the whole genome-based taxonomic analysis via the Type (Strain) Genome Server (TYGS) platform (https://tygs.dsmz.de). The type-based species clustering using a 70% dDDH radius around each of the type strains was used, while subspecies clustering was done using a 79% dDDH threshold as previously introduced [31 .32]. Different species are identified by color.

Table S3. Average Nucleotide Identify (ANI) and Alignment fraction (AF) values for *Frankia* isolates from Lineage 1c.

**Genome 1:**	***Frankia* sp. CgIM4**				***Frankia* sp. CgIS1**				***Frankia* sp. CcI156**			
**Genome 2**	**ANI1->2**	**ANI2->1**	**AF1->2**	**AF2->1**	**ANI1->2**	**ANI2->1**	**AF1->2**	**AF2->1**	**ANI1->2**	**ANI2->1**	**AF1->2**	**AF2->1**
Frankia casuarinae CcI3	99.6022	99.5923	88.892	81.741	99.7456	99.7352	86.682	83.416	99.6292	99.616	86.322	84.046
Frankia sp. CcI6	99.6454	99.643	92.271	86.215	99.9614	99.9612	97.93	95.528	99.8312	99.8308	97.895	96.697
Frankia sp. BMGG5.23	99.5058	99.5055	85.807	82.762	99.6131	99.6134	85.64	86.289	99.4913	99.4912	85.595	87.314
Frankia casuarinae BR	99.5535	99.5528	90.748	87.395	99.8527	99.8528	97.103	97.62	99.7188	99.7187	95.716	97.43
Frankia sp. KB5	99.2952	99.2959	82.566	77.897	99.4352	99.4372	77.673	76.548	99.3144	99.3158	77.792	77.602
Frankia casuarinae Cg70.9	99.18	99.18	75.87	79.13	99.332	99.3391	71.154	77.628	99.2176	99.2209	71.396	78.837
Frankia casuarinae Thr	99.6136	99.6087	89.029	84.357	99.7526	99.7473	89.519	88.739	99.6251	99.6198	89.327	89.615
Frankia sp. Allo2	99.5755	99.5752	92.187	86.895	99.859	99.859	97.694	96.221	99.7278	99.7278	97.362	97.095
Frankia sp. CeD	99.4306	99.4309	84.713	85.282	99.5505	99.5503	84.772	89.172	99.4119	99.4119	84.603	90.099
Frankia sp. CcI156	99.537	99.537	92.53	87.506	99.8192	99.8196	98.094	96.89	-	-	-	-
Frankia sp. CgIS1	99.6532	99.653	91.741	87.839	-	-	-	-	99.8196	99.8192	96.89	98.094
Frankia sp. CgIM4	-	-	-	-	99.653	99.6532	87.839	91.741	99.537	99.537	87.506	92.53

ANI, Average Nucleotide Identity; AF, Alignment Fraction

ANI>=96.5 and AF>=60 values represents same species
